# Comparisons of foot anthropometry and plantar arch indices between German and Brazilian children

**DOI:** 10.1186/s12887-015-0321-z

**Published:** 2015-02-12

**Authors:** Isabel CN Sacco, Andrea N Onodera, Kerstin Bosch, Dieter Rosenbaum

**Affiliations:** Physical Therapy, Speech and Occupational Therapy Department, School of Medicine, University of São Paulo, São Paulo, Brazil; Gait Lab, Social Pediatric Centre, Children’s Clinic, St.-Vincenz-Hospital, Coesfeld, Germany; Movement Analysis Lab, Institute of Experimental Musculoskeletal Medicine (IEMM), University Hospital Münster, Münster, Germany; Funktionsbereich Bewegungsanalytik, Institut für Experimentelle Muskuloskelettale Medizin, Zentrum für Muskuloskelettale Medizin, Universitätsklinikum Münster, Albert-Schweitzer-Campus 1, Gebäude D 3, D-48149 Münster, Germany

**Keywords:** Longitudinal plantar arch, Body weight and measures, Child, Preschool, Feet

## Abstract

**Background:**

Nowadays, trades and research have become closely related between different countries and anthropometric data are important for the development in global markets. The appropriate use of anthropometry may improve wellbeing, health, comfort and safety especially for footwear design. For children a proper fit of footwear is very important, not constraining foot growth and allowing a normal development. The aim of this study was to compare the anthropometric characteristics of German and Brazilian children’s feet from 3 to 10 years of age.

**Methods:**

We compared five indirect measures of two databases of children's feet. Forefoot, midfoot and rearfoot widths were measured in static footprints and the Chippaux-Smirak and Staheli indices of the longitudinal arch were calculated.

**Results:**

Brazilian children showed a significantly narrower forefoot from 5 to 10 years, wider rearfoot from 3 to 4 years, wider midfoot for 4 year-olds and narrower midfoot for 10 year-old children. Nevertheless, the Chippaux-Smirak and Staheli indices showed no group differences. The only exception was for 4 year-old Brazilian children who showed a higher Chippaux-Smirak index compared to German children (48.4 ± 17.7%; 42.1 ± 13.8%).

**Conclusions:**

Our study revealed anthropometric differences in absolute forefoot and rearfoot widths of German and Brazilian children, but a similar longitudinal arch development. At 4 years of age, Brazilian children present a foot anthropometry similar to the 3 year-olds and develop the plantar longitudinal arch from 4 to 5 years more rapidly when compared to German children.

## Background

Due to world globalization, trades and research have become closely related between different countries. Anthropometric data are important for product design and development in global markets. Appropriate use of anthropometric measures may improve wellbeing, health, comfort and safety, especially for footwear design.

In normal human growth, foot shape and proportions change progressively due to several aspects, however a key factor for foot development are mechanical stresses during bipedal locomotion. Especially for children, a proper fit of footwear is important, not constraining foot growth and allowing a normal development. Therefore, the lasts used in children’s footwear industry should fit the foot morphology according to user’s foot dimensions to produce comfortable shoes, avoiding subsequent foot deformities for the rest of their lives [1,2].

Not only genetic inheritance, but also differences in the environment, socio-economic development, ethnicities and cultures influence the demographic and anthropometric characteristics. Mauch *et al.* [2] compared the foot morphology between Australian and German children and observed longer feet in Germany. Actually the authors were expecting longer feet in populations from warmer climate due to the habitual use of open footwear and barefoot walking. One potential explanation for this finding was the mixed ethnic population in Australia. In June 2006, the proportion of the Australian population born overseas was 24% with a high percentage of Asian-born immigrants, while the German population is predominantly Caucasian with only 8% classified as non-European.

Comparing urban and rural populations, Kusumoto [3] showed that rural Philippine children (7 to 13 years) were shorter and lighter than urban Japanese children. These differences were related to nutritional and life styles factors. However, foot width and circumference did not differ. Ashizawa *et al.* [4] attributed foot morphology differences among Javanese, Philippine and Japanese adults to the habitual footwear use.

Although there is high variability among foot anthropometry due to ethnicity, culture and daily habits [2-6], the footwear industries still do not vary the last dimensions when exporting products to different continents [3].

Despite the number of studies investigating foot anthropometry in Asia [3-5], Europe [2,6,7] and North America [8], a country like Brazil with continental extent and a unique mixture of ethnic backgrounds still lacks foot anthropometry standards, forcing its footwear industries to use anthropometric data from other nations, mostly from Europe, without knowing if it is appropriate. This study aimed to compare anthropometric characteristics of children’s feet from 3 to 10 years of age between German and Brazilian populations, which are known to differ in climate, lifestyle and ethnicity which could potentially influence foot growth and development.

## Methods

### Anthropometric databases

Two anthropometric databases were used [7,9]. The German database consisted of 94 healthy German children of both sexes from the onset of walking to 10 years followed over 9 years in a longitudinal approach (Table 1). The children were assessed 17 times during 9 consecutive years by a trained expert assessor with many years of experience in biomechanical assessments to avoid systematic bias throughout the longitudinal measurements. For our study, we took the yearly appointments from 3 to 10 year-old German children which were part of this longitudinal study on the development of children’s feet (*Kidfoot Münster*). The Brazilian database consisted of 391 healthy children of both sexes from 3 to 10 years recruited from Children’s Centers in a cross-sectional study (Table 1). Exclusion criteria for both countries were orthopedic, neurologic, systemic diseases or pre-term births. Height and body mass were recorded at every appointment.

**Table 1:** **Means and standard deviations of height, body mass and percentage of males by age for the German and Brazilian databases**

	**n**		**% of boys**		**Height (cm)**		**Body mass (kg)**		**Body mass index (kg/m** ^**2**^ **)**		Median (German Normative Data)**	
**Age groups**	**Brazil**	**Germany**	**Brazil**	**Germany**	**Brazil**	**Germany**	**Brazil**	**Germany**	**Brazil**	**Germany**	***Boys***	***Girls***
**3**	32	94	37.5	46.8	96.1 (3.6)	99.0 (4.0)*	15.1 (1.8)	15.6 (1.6)	16.5 (1.4)	15.9 (1.3)	*15.6*	*16.6*
**4**	73	92	45.2	46.8	105.0 (4.5)	106.8 (4.3)*	18.0 (3.0)	18.0 (2.0)	16.3 (2.2)	15.8 (1.3)	*15.5*	*16.4*
**5**	62	92	33.9	47.7	111.4 (5.3)	114.2 (4.9)*	19.7 (2.7)	20.5 (2.4) *	15.8 (1.8)	15.7 (1.4)	*15.4*	*16.5*
**6**	74	88	48.6	50.0	115.5 (5.4)	121.0 (5.2)*	21.7 (3.1)	23.4 (2.7)*	16.2 (1.9)	15.9 (1.3)	*15.5*	*16.6*
**7 and 8**	32	85	43.7	46.7	125.5 (6.3)	129.5 (6.3)*	25.3 (3.9)	26.8 (3.4)*	16.1 (1.8)	16.0 (1.5)	*15.8*	*17.2*
**9**	83	54	54.2	47.3	136.0 (6.7)	138.8 (6.7)*	31.9 (5.5)	32.2 (4.4)	17.2 (2.2)	16.7 (1.9)	*16.4*	*18.1*
**10**	35	51	62.9	45.8	141.3 (8.0)	145.6 (6.7)*	36.7 (9.5)	36.6 (5.2)	18.2 (3.6)	17.2 (2.1)	*16.9*	*18.7*

*Statistically higher values between German and Brazilian databases [t-test (parametric data) or Mann-Whitney test (non-parametric data), p < 0.05].

**normative values from Kromeyer-Hauschild et al. [12].

Local Ethics Committees approved each investigation and parents signed informed consent forms. In Germany, the “Ethikkommission der Ärztekammer Westfalen-Lippe und der Medizinischen Fakultät der Westfälischen Wilhelms-Universität Münster” approved the project (Reference No. 1-V-Ros). The procedures in Brazil were approved by the University of Sao Paulo Institutional Review Board for Human Subjects, and a written informed consent was obtained prior to the children’s participation by their parents or guardians.

By selecting the appropriate ‘whole-year’ measurements from the available 17 longitudinal appointments, the German database was divided into age groups of 3 to 10 years to make it comparable to the Brazilian database, which was already divided into age subgroups. Due to the small number of individuals of ages 7 and 8 years in the Brazilian database, they were grouped in both databases to form an age subset from 7 to 8 years.

### Anthropometric data and statistical analysis

In both countries, static foot shape parameters of both feet were acquired with Harris mat footprints in bipedal stance and evenly distributed weight-bearing. Forefoot, midfoot and rearfoot widths of the right side were analyzed. Since body height could affect foot dimensions [7], and German children were significantly taller than Brazilians (Table 1), the foot widths were normalized by the height of each child.

**Figure 1:**
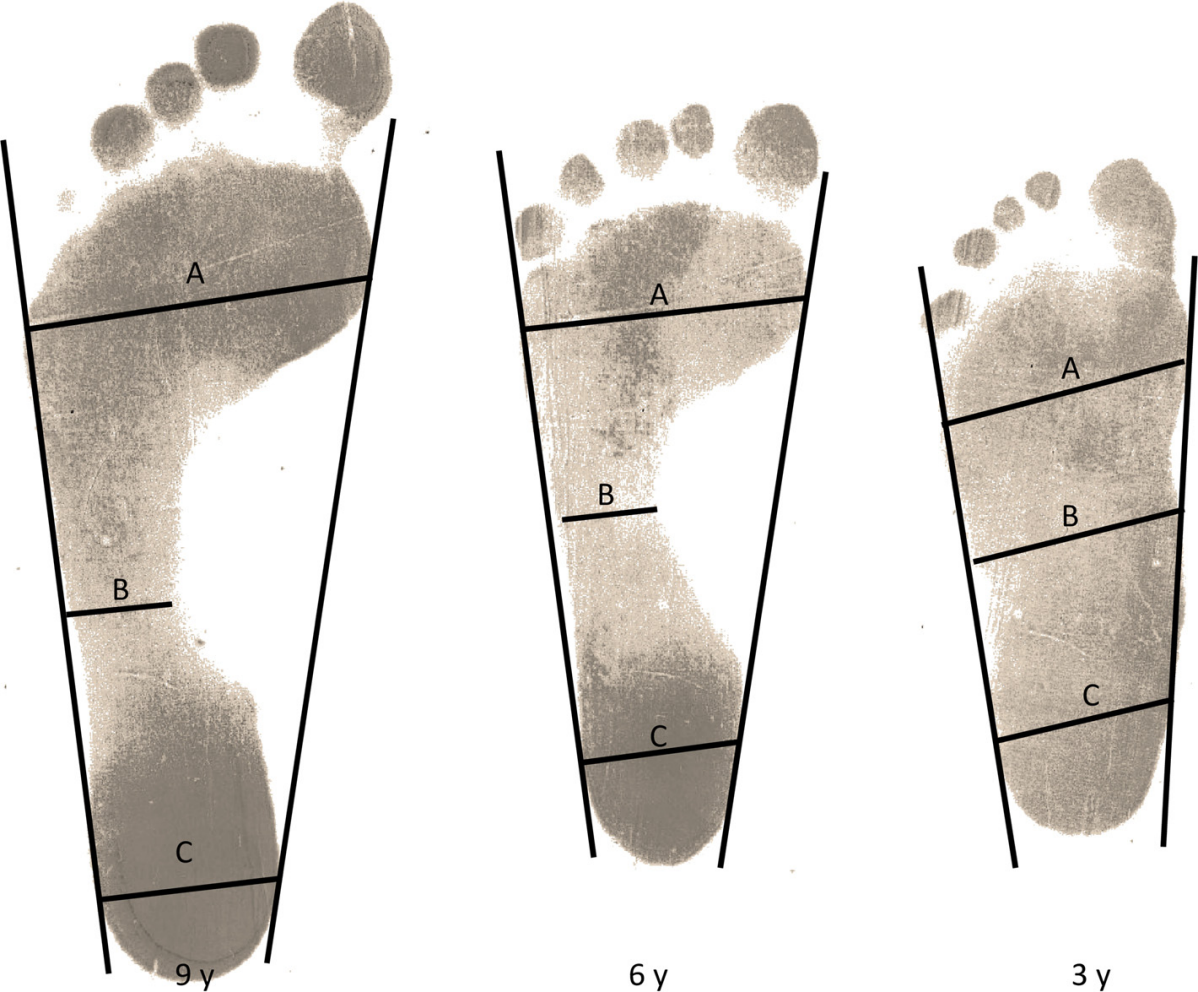
**Representative illustration of footprints of 3 age groups and the lines used for Chippaux-Smirak and Staheli Indices calculations.** ‘**A**’ represents the widest width of forefoot, ‘**B**’ represents the narrowest width of midfoot and ‘**C**’ represents the widest width of rearfoot.

We also compared the longitudinal plantar arch development between populations using the Chippaux-Smirak [10] and Staheli Indices [11] calculated from the right footprints. The Chippaux-Smirak Index was calculated as the ratio between the smallest length of midfoot (Figure 1B) and the largest length of the metatarsal heads regions (Figure 1A). The Staheli Index was calculated as the ratio between the smallest length of the midfoot (Figure 1B) and the largest length of the heel (Figure 1C).

The normal distribution (Kolmogorov-Smirnov test), and homogeneity of variances (Levene test) were tested for all data and independent t-tests were used to compare normally distributed data, and non-parametric variables were compared by Mann-Whitney U-tests with Statistica software (version 10). The alpha level was set to 5%.

For the sample sizes evaluated in both groups (n = 391 versus n = 94) and the statistical comparison between the two groups using t-tests or Mann Whitney tests, we achieved a power of 0.832, for a small effect size (d = 0.20) and an alpha error of 0.05.

## Results

German children showed a significantly wider normalized forefoot than Brazilians from 5 to 9 years (Tables 2 and 3). The normalized midfoot width was similar between groups except for the 4 year-olds (Tables 2 and 3). Brazilian children of 4 years showed a wider midfoot than Germans. Brazilian children had a wider rearfoot at the age of 4 years (Tables 2 and 3). There were no significant differences in both arch indices for all ages, except for the 4 year-old group, where Brazilian children showed a higher Chippaux-Smirak index (lower plantar arches) (Tables 2 and 3).

**Table 2:** **Means and standard deviations of forefoot (FFW), midfoot (MFW) and rearfoot widths (RFW) normalized by children’s height (%), Chippaux-Smirak Index (CSI) and Staheli’s Index (SI) of the Brazilian and German populations**

**Age groups**	**FFW (%)**		**MFW (%)**		**RFW (%)**		**CSI**		**SI**	
	**Brazil**	**Germany**	**Brazil**	**Germany**	**Brazil**	**Germany**	**Brazil**	**Germany**	**Brazil**	**Germany**
**3**	5.89 (0.42)	5.84 (0.35)	2.85 (0.95)	2.72 (0.98)	3.36 (0.30)	3.27 (0.26)	48.8 (16.0)	46.4 (16.1)	0.83 (0.24)	0.84 (0.28)
**4**	5.76 (0.47)	5.84 (0.36)	2.80 (1.05)*	2.44 (0.93)	3.38 (0.27)*	3.13 (0.28)	48.4 (17.5)*	41.5 (15.0)	0.81 (0.28)	0.78 (0.29)
**5**	5.53 (0.46)	5.86 (0.32)*	2.19 (1.10)	2.28 (0.83)	3.13 (0.29)	3.15 (0.23)	39.2 (20.2)	38.0 (12.9)	0.68 (0.32)	0.72 (0.25)
**6**	5.61 (0.32)	5.77 (0.35)*	2.08 (1.02)	2.06 (0.82)	3.13 (0.27)	3.13 (0.21)	37.6 (19.0)	35.3 (13.3)	0.66 (0.31)	0.65 (0.26)
**7 and 8**	5.47 (0.43)	5.67 (0.35)*	1.77 (0.85)	1.70 (0.90)	3.09 (0.29)	3.03 (0.21)	32.1 (14.5)	29.6 (15.4)	0.57 (0.25)	0.55 (0.29)
**9**	5.46 (0.36)	5.67 (0.41)*	1.65 (0.96)	1.73 (0.88)	3.03 (0.28)	3.01 (0.27)	30.4 (17.0)	30.1 (15.0)	0.54 (0.29)	0.57 (0.29)
**10**	5.47 (0.32)	5.60 (0.37)	1.41 (0.74)	1.68 (0.83)	2.94 (0.32)	2.95 (0.24)	26.0 (13.4)	29.2 (14.9)	0.46 (0.23)	0.55 (0.28)

*Statistically higher values between German and Brazilian databases [t-test (parametric data) or Mann-Whitney test (non-parametric data), p < 0.05].

**Table 3:** **Means (standard deviation) of absolute width measurements of the forefoot, midfoot and rearfoot of Brazilian and German children**

**Age groups**	**Forefoot width (mm)**		**Midfoot width (mm)**		**Rearfoot width (mm)**	
	**Brazil**	**Germany**	**Brazil**	**Germany**	**Brazil**	**Germany**
**3**	56.9 (4.7)	58.0 (3.7)	27.7 (9.1)	26.9 (9.5)	33.1 (3.6)	32.5 (2.8)
**4**	60.4 (6.0)	62.3 (4.0)	29.4 (11.1)	26.0 (9.7)	35.2 (3.3)	33.5 (3.2)
**5**	61.5 (6.5)	66.9 (4.0)	24.2 (12.9)	25.5 (8.9)	35.0 (3.9)	36.3 (2.9)
**6**	65.2 (4.8)	69.8 (4.6)	24.5 (12.6)	24.8 (9.7)	36.2 (3.5)	37.8 (3.0)
**7 and 8**	68.4 (5.2)	73.4 (4.4)	22.0 (9.8)	21.8 (11.4)	38.4 (3.1)	39.2 (3.0)
**9**	74.6 (5.8)	78.6 (5.2)	22.9 (13.2)	23.9 (12.2)	41.3 (4.4)	41.7 (3.3)
**10**	77.4 (6.3)	81.3 (5.1)	20.3 (10.3)	23.9 (12.6)	41.3 (5.1)	42.9 (4.1)

## Discussion

The results showed that German children had a wider forefoot from 5 to 9 years and a narrower rearfoot for 4 years compared to Brazilian children. Nevertheless, the longitudinal arch development was not affected and develops similarly for both populations, except for the 4 year-old Brazilian children who presented lower arches.

German children were taller than Brazilians in all age groups and heavier from 5 to 8 years. However, there were no statistical differences for body mass index, which was classified as normal for all age groups from both countries as compared to normative data from a large cohort of over 34000 children in Germany [12]. Although Brazil is not considered an extremely poor country, socio-economic differences between these two nations could be a reason for the higher height and body mass of German children. According to the WHO, Germany had in 2012 a total health expenditure per capita of 4617 US$ [13], while Brazil had only 1109 US$ [14]. From 2003 to 2006, the prevalence of low height and weight in German children under 5 years was 1.3% [15], in Brazilian children 7.1% [16]. Kusumoto [3] attributed height and body weight differences of Philippines and Japanese children to specific diets in the countries. Children from Tokyo increased their protein intake, especially after the end of the World War II, compared to the Philippines. The traditional Brazilian dish consists of a mixture of rice, beans [17], sometimes with salad and meat, while German meals often consist of meat and potatoes [18]. Brazil has seen a remarkable increase in the consumption of animal products, although the levels are still well below North America and Germany [19]. These differences in daily diet (especially protein intake) could be related to the differences in children’s growth.

Brazilian children had a wider rearfoot during the early ages (3 and 4 years), a narrower forefoot for 5 to 10 years, a wider mid-foot for 4 year-olds, and a narrower mid-foot for 10 year-olds. These differences demonstrate the distinct morphology of German and Brazilian feet. Footwear manufacturers supplying both countries should consider these results. The common use of European lasts in Brazilian shoe companies might affect children’s shoe fitting. Brazil is a country of ethnic diversity due to its history. Brazil used to be a Portuguese colony until 190 years ago, with plantations that involved many African immigrants. Indians, Africans and Europeans could be the greatest influence in the anthropometric characteristics of Brazilian people, while the German population is predominantly Caucasian with only 8% of the population classified as non-European [2]. Hawes *et al.* [8] showed that African feet are slightly narrower in the forefoot and wider in the rearfoot when compared to Caucasian feet of similar length. This may explain different widths between German and Brazilian children. However, the Brazilian population origins did not affect the maturation of the medial longitudinal arch. Echarri and Forriol [20] found a higher proportion of flatfeet in urban Congolese children compared to children from rural regions due to the habitual footwear use. It was expected that the tropical climate of Brazil, with habitual use of open sandals and flip-flops could lead to a faster maturation of the longitudinal arch. However, the plantar arch development was similar in both populations. The urban characteristic of both populations might have contributed to the similar arch development. The difference between longitudinal arch development in German and Brazilian children was restricted to the 4 year-olds, where Brazilian children had an arch maturation similar to 3 year-olds, while German children presented a gradual and progressive development of the arch.

The lack of foot length information is one limitation of this study. From the German database, it was possible to recover foot length; however, we missed this parameter in the Brazilian database. Although it is not uncommon to normalize the foot measures by the foot length [2], some authors have already reported significant and relevant association between body height and foot length [5,7]. Therefore, we chose to normalize the foot measures by the height. We do believe it did not compromise the analysis and the subsequent results. A further difference was that the Brazilian and German data were collected with different experimental designs, i.e. cross-sectional versus longitudinal approach. In order to avoid systematic error in the longitudinal data acquisition, measurements were carried out with the same equipment and by the same expert with many years of experience in biomechanical assessments. However, even with this design, there is still a possibility that the results of the present study could be affected by the different methodologies. Future studies could measure more parameters, like foot length, ball of foot circumference, arch length, which are also important measurements for the footwear industry.

## Conclusions

German children tend to have a wider forefoot from 5 to 9 years and narrower rearfoot in early age (4 years) compared to Brazilian children. The differences in absolute widths do not affect the longitudinal plantar arch, which develops comparably in both populations in most of the ages. The only exception is for the 4 year-old children, where Germans develop the longitudinal plantar arch gradually from 3 to 10 and Brazilian children of 4 years have their foot anthropometry similar to the 3 year-old children and after that develop their plantar arch rapidly from 4 to 5 years. Therefore, the use of European lasts in Brazilian shoe companies would not ensure a good fit for Brazilian children shoes.
